# Microbial degradation of Procion Red by *Pseudomonas stutzeri*

**DOI:** 10.1038/s41598-021-82494-9

**Published:** 2021-02-04

**Authors:** Sweta Parimita Bera, S. K. Tank

**Affiliations:** Department of Biosciences, Veer Narmad South Gujarat University, Surat, Gujarat India

**Keywords:** Microbiology, Environmental sciences

## Abstract

The bacterium *Pseudomonas stutzeri* SPM-1, obtained from textile wastewater dumping sites of Surat, Gujarat was studied for the degradation of the textile azo dye Procion Red—H3B. The optimization was carried on the phenanthrene enrichment medium followed by exposing it to variable environmental factors and nutritional sources. The complete decolorization of dye (50 mg/L) happened within 20 h of incubation at pH 8 and temperature 32 ± 0.2 °C under microaerophilic conditions. Decolourization was monitored with the shifting of absorbance peak in UV–Vis spectrophotometry and HPLC analysis. The physicochemical studies of effluent before and after the treatment revealed 85%, 90%, and 65% decline in BOD, COD, and TOC levels. The strain showed significant activities of azoreductase (95%), laccase (76%), and NADH-DCIP reductase (88%) at 12 h, 10 h, and 8 h of growth respectively indicating evidence for reductive cleavage of the dye. The changes in the functional groups were confirmed by the presence of new peaks in FT-IR data. GC–MS analysis helped in recognizing the degraded dye compounds thus elucidating the proposed pathway for degradation of Procion Red—H3B. The potential of the bioremediation process was concluded by a phytotoxicity test using two plants, *Vigna radiata* and *Cicer arietinum*. Our study demonstrates that the strain *Pseudomonas stutzeri* SPM-1 has rapid decolorization efficiency and holds a noteworthy perspective in industrial application for textile wastewater treatment.

## Introduction

Every year nearly 7 × 10^5^ metric tons of dye is produced worldwide, out of which azo dyes account for 70%. About 50–90 g of dyestuff and nearly 100–150 L of water is required to color merely 1 kg of cotton^1^. The effluent generated during the textile processing is majorly composed of dye molecules that are lost during the dye application processes. As the demand for vibrant fabrics kept growing, synthetic dyes such as reactive azo dyes have been extensively used as commercial textile dyes. Reactive azo dyes are used mainly for properties like efficient wet fastness, effortless application, low energy requirement, and availability of bright varieties of color shades. Procion Red—H3B is one such extensively used commercial textile azo dye. The dye molecule has large molecular weight and is highly difficult to degrade. Additionally, the dye is toxic, carcinogenic, and hazardous to both the environment and humans. When discharged into the water bodies this leads to bioaccumulation resulting in interruption of the complete ecosystem^2^.

There are many physicochemical processes already available for the treatment of dyes in the textile effluent. A number of these methods are useful but are practically expensive as they generate large amounts of chemical sludge. The disposal of this highly toxic chemical sludge is detrimental to the landfills and contributes heavily to the cost of processing^3^. Biological treatment is one of the safest and environmentally friendly approaches that serve as a real hope. The application of bioremediation in degrading the dye molecules is proficient, cost-effective, and nontoxic to the environment. The survival and adaptability of microorganisms during the treatment processes determines the effectiveness of these treatment systems^4^. But dye house effluents usually contain azo dyes that are highly resistant to biological treatment. The presence of N=N bonds and other possible groups, like the sulphonic group makes the dye difficult to be degraded by the microorganisms^5^.

Bacterial strain when provided with optimum environmental conditions and a nutritional source, does wonder in the process of bioremediation. Many reports have demonstrated the role of various environmental as well as nutritional factors responsible for accelerating the complete mineralization of dyes during bioremediation treatment^6^. The pH of the medium has a key role in the efficacy of dye decolorization; it helps in the transport of dye molecules across the cell membrane^7^. The majority of research reported suggests that the optimum pH for decolourization by the bacteria is usually between pH 6.0 and 10.0. Similarly, temperature that is little above normal is good for decolorization, but if increased to a higher temperature it can lead to denaturation of the bacterial enzymes resulting in a decline of the decolorization rate. Higher dye concentration tends to inhibit bacterial growth as they increase the toxicity of the medium^8^. The microbes with competent metabolic mechanisms help in breaking the complex dye molecules into metabolites that are safe to be discarded into the environment^9^. The strain *Pseudomonas stutzeri* SPM-1 isolated from the textile effluent dumping site displayed natural transformation abilities. Biochemical analysis of the decolorization process exhibited the strain's ability to enzymatically degrade the dye molecules. Previous studies on *Pseudomonas stutzeri* suggest their physiological characters like denitrification, natural transformation, and capability to degrade environmental contaminants^7^. In this study, the strain was optimized tobring about maximum possible degradation of the reactive azo dye. The strain displayed exceptional decolorization, and degradation abilities within the span of 20 h. Thus, it was concluded that the strain exhibits a high possibility for bioremediation of textile effluents containing reactive azo dyes. Looking into the current problem of wastewater treatment faced by the textile industries, this research study can serve as a potential bio remedial application.

## Results

### Optimization of decolorization conditions

**Figure 1:**
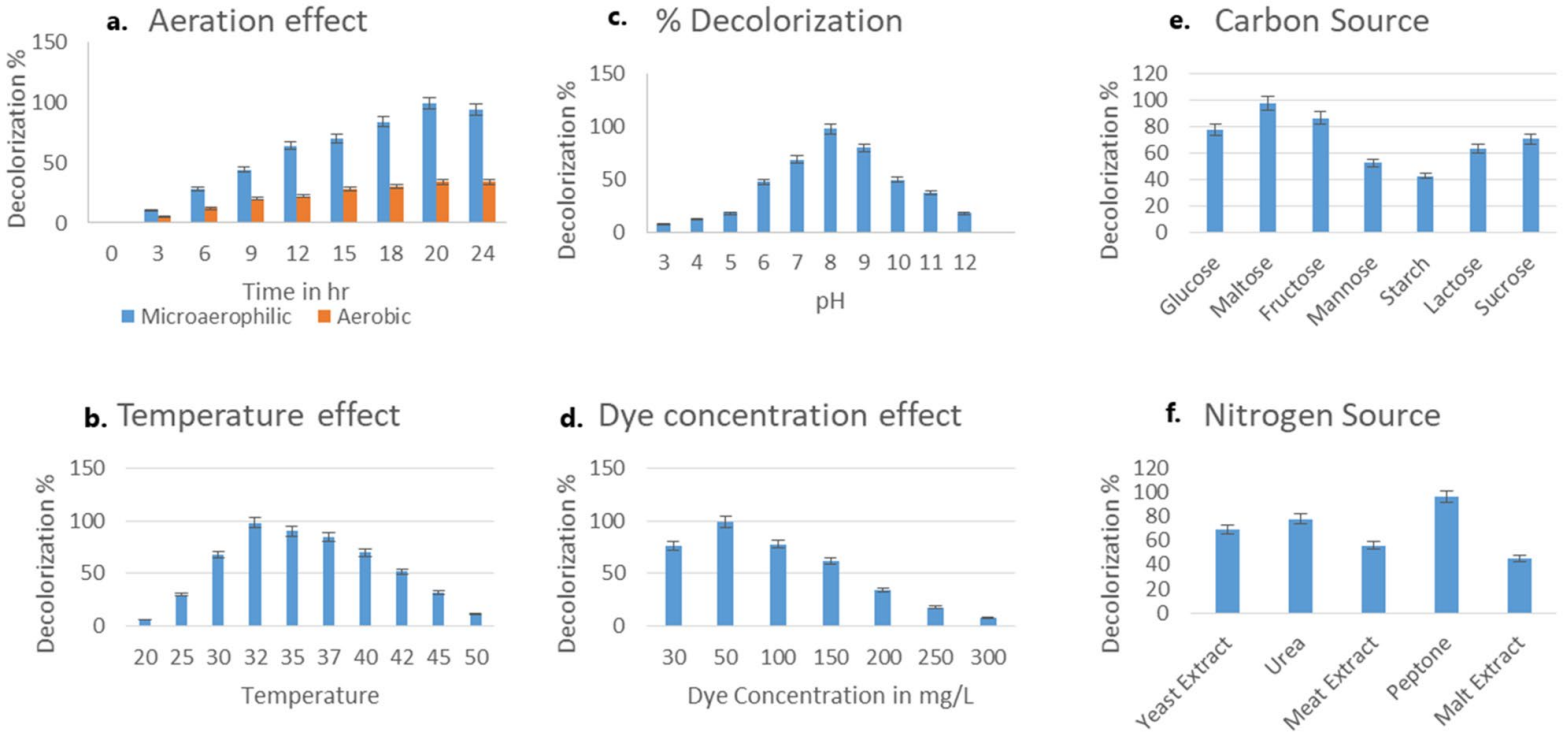
Effect of Environmental and nutritional factor on decolourization %. (**a**) Effect of aeration, (**b**) Effect of temperature, (**c**) Effect of pH, (**d**) Effect of dye concentration, (**e**) Effect of carbon sources, and (**f**) Effect of nitrogen sources.

In this study, the bacterial strain was subjected to a range of pH; and it was found that decolourization percentage was optimal at pH 8 and temperature 32 ± 0.2 °C under microaerophilic condition. The decolourization percentage was remarkably reduced at lower pH (3–5) and higher pH (9–12). This signifies the inhibition of decolourization activity at extreme pH conditions. The strain *P. stutzeri* SPM-1 showed optimum colour removal activity within 20 h of incubation in microaerophilic condition. Moreover, when the temperature was increased (37°, 40°, and 50 °C) or decreased (20 °C) it resulted in significant decline in the decolorization performance. The strain was exposed to different dye concentrations ranging from 50 to 250 mg/L and the maximum decolourization was observed at 50 mg/L. The microbial degradation of azo dye is difficult without any supplement of carbon or nitrogen sources as the dye itself is deficient in carbon sources^10^. The strain exhibited optimum decolourization of dye in the presence of carbon and nitrogen source as co-substrate. The different carbon sources tested in the present study are glucose, maltose, fructose, mannose, starch, lactose, sucrose (10 g/L). Maltose was found to be better carbon source showing maximum decolourization (98%) while glucose (78%), fructose (87%), mannose (53%), starch (43%), lactose (64%) and sucrose (71%) showed decolourization of the dye respectively. The nitrogen sources analysed in the present study were yeast extract, urea, meat extract, peptone and malt extract (3 g/L). Peptone with 96% decolourization showed extra decolourization percentage among all the other nitrogen sources. The decolourization percentage by yeast extract, urea, meat extract, and malt extract were 69%, 78%, 56% and 48% respectively. Figure 1 depicts the decolourization percentage at various environmental and nutritional parameters.

### Decolorization of textile azo dye procion red—H3B

**Figure 2:**
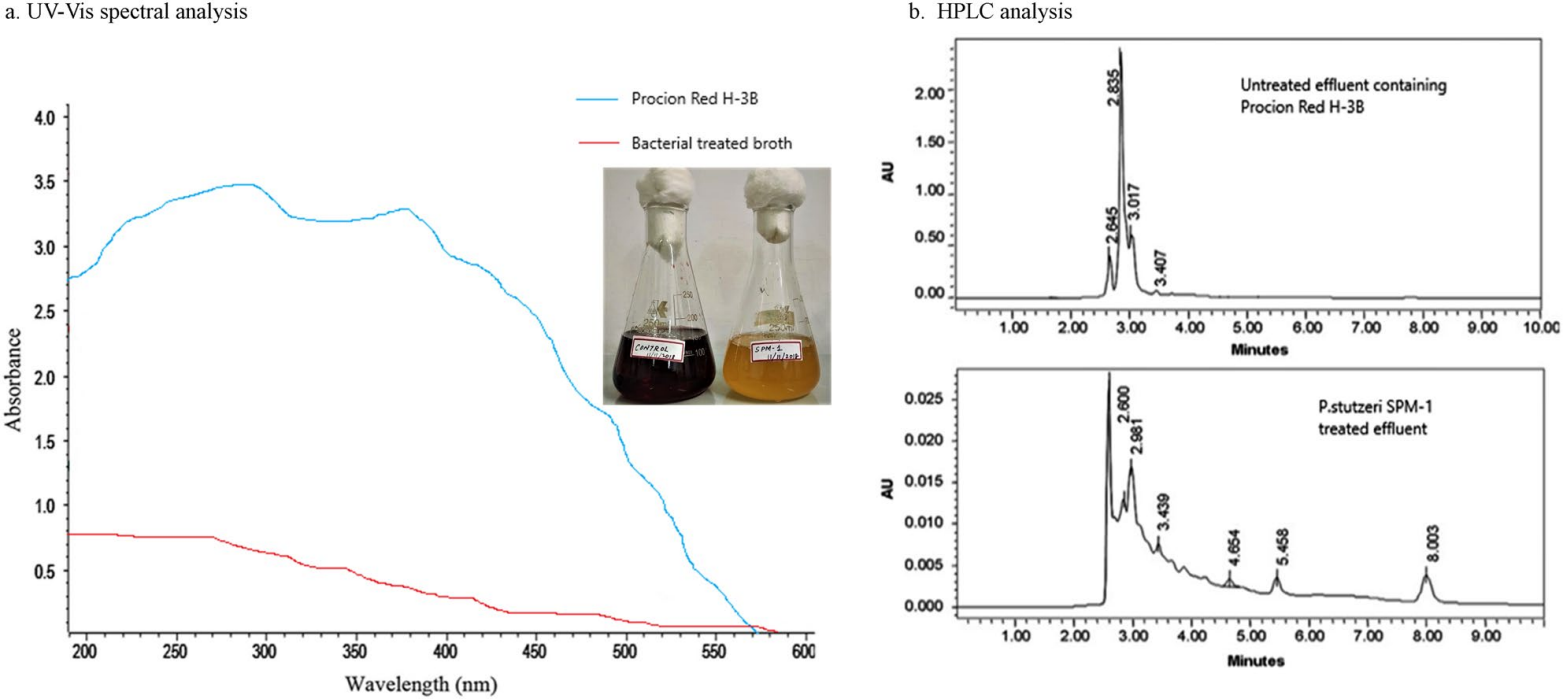
Analysis of untreated and *P. stutzeri* SPM-1 treated effluent containing Procion Red H-3B by (**a**) UV–Vis spectral analysis and (**b**) HPLC analysis.

The dye decolourization ability of the strain *P. stutzeri* SPM-1 was studied on phenanthrene mineral medium. This specialized mineral salt medium containing phenanthrene helps the growth medium in co-metabolic degradation of polycyclic aromatic hydrocarbon compounds^11^. The initial decolourization of Procion Red—H3B by the strain *P. stutzeri* SPM-1 under microaerophilic conditions showed encouraging results. The confirmation was carried by UV–Vis spectral analysis (200–600 nm). Maximum decolourization of the dye was observed at 585 nm. Although, the bacterial growth is significantly affected by the aeration procedure involving oxygen, *P. stutzeri* SPM-1 exhibited excellent decolourization under microaerophilic condition. The strain was ableto bring complete decolourization (50 mg/L) within 20 h at 32 ± 0.2 °C. The analysis for aerobic condition showed only 20% decolourization at 20 h and 30% at 24 h. Figure 2a shows the UV–Vis spectral analysis of untreated and *P. stutzeri* SPM-1 treated effluent containing Procion Red H-3B. Microaerophilic condition clearly demonstrated higher decolourization rate of the dye Procion Red—H3B by the strain *P. stutzeri* SPM-1.

### Study of dye mineralization

The physico-chemical studies of effluent before and after the treatment revealed significant variations. The bacterial culture was able to reduce the pollutants to a great extent. The mineralization of the dye after treatment with the bacterial strain was examined by the rate of decreases in BOD, COD and TOC contents. The environmental conditions for the degradation activity was pre-set at pH 8.0, temperature 32 ± 0.2 °C, dye concentration of 50 mg/L, with microaerophilic incubation. The result showed 85%, 90% and 65% decline in BOD, COD and TOC level respectively after 20 h of incubation. The physico-chemical analysis of the effluent before and after the bacterial treatment are summarised in Table 1.

**Table 1:** Physico-chemical analysis of the effluent before and after the bacterial treatment.

Parameter	Untreated (control)	Bacterial treated (20 h)	Reduction %	DoE Standards (2015)
Colour	Reddish brown	slight yellowish	–	–
pH	10 ± 0.0	8 ± 0.20		6.5–9
BOD(mg/L)	7969 ± 145.23	1195.5 ± 120.65	85	150
COD(mg/L)	12,321 ± 85.65	1232.95 ± 38.56	90	200
TDS(mg/L)	953 ± 5.01	286.09 ± 2.50	70	200
TSS(mg/L)	950 ± 6.90	238.19 ± 3.45	75	100
TOC(mg/L)	1281 ± 7.02	449.05 ± 5.0	65	250

Values are mean of three experiments ± standard deviation (SD).

### Study of enzyme activity

According to the results of enzymatic activity shown by the strain *P. stutzeri* SPM1, it was noted that the strain possesses azo reductase, laccase and NADH-DCIP reductase enzyme system in control cells. The strain displayed remarkable role in the degradation at the initial stages and decreased gradually at 18 h of incubation. The colour reduction was initiated at 8 h and reached maximum decolourization at 20 h. The decolourization study was conducted for 48 h but the results were evident at the initial 20 h. The amount of azoreductase increased from 0.194 ± 0.05 mL^−1^ 1 min^−1^ to 0.378 ± 0.03 mL ^−1^ min^−1^ which was 95% increase in the enzyme content in 12 h of incubation. In the laccase activity an increase of 76% was noted with 0.198 ± 0.03 mL^−1^ min^−1^ at the initially stage and 0.348 ± 0.02 mL^−1^ min^−1^ after decolourization at 10 h, which was comparatively lesser than the azoreductase activity. About 88% increase was observed in the NADH-DCIP reductase activity after 8 h of incubation. The amount before decolourization was 12 ± 1.50 mL^−1^ min^−1^ and rose to nearly 22.56 ± 1.85 mL^−1^ min^−1^ after decolourization. Table 2 depicts the enzyme activity during decolourization of dye Procion Red—H3B by the strain *P. stutzeri* SPM1.

**Table 2:** Enzyme activity during decolourization of dye Procion Red—H3B by strain *P. stutzeri* SPM1.

Enzymes	Control cells (0 h)	After decolourization (15 h)
Azo reductase^1^	0.194 ± 0.05	0.378 ± 0.03*
Laccase^2^	0.198 ± 0.03	0.348 ± 0.02*
NADH-DCIP reductase^3^	12 ± 1.50	22.56 ± 1.85*

Values are mean of three experiments ± standard error of mean (SEM), significantly different from control cells at *P < 0.001 by one-way analysis of variance (ANOVA) with Tukey–Kramer comparison test.

^1^ μM of methyl red reduced min^−1^ mL of enzyme^−1^ mg of protein^−1^.

^2^ μM of ABTS oxidized min^−1^ mL of enzyme^−1^ mg of protein^−1^.

^3^ μM of DCIP reduced min^−1^ mL of enzyme^−1^ mg of protein^−1^.

### Study of biodegradation of the dye Procion Red H-3B by HPLC

The HPLC analysis was conducted using effluent containing the dye Procion Red H-3Bas the control. It exhibited the occurrence of one major peak at retention time of 2.835 min and three minor peaks at retention times of 2.645, 3.017, and 3.407 min. The disappearance of peaks after the removal of dye was seen in the case of treated effluent sample.The development of entirely different three major peaks at retention times of 2.600, 2.981, and 3.439 min and three minor peaks at retention time of 4.654, 5.458 and 8.003 min were observed after the decolourization. The emergence of new minor peaks and desertion of the most important peak in the decolorized dye products elution profile sustain the biodegradation of Procion Red—H3B. Figure 2b shows the results of HPLC analysis.

### FT-IR analysis

**Figure 3:**
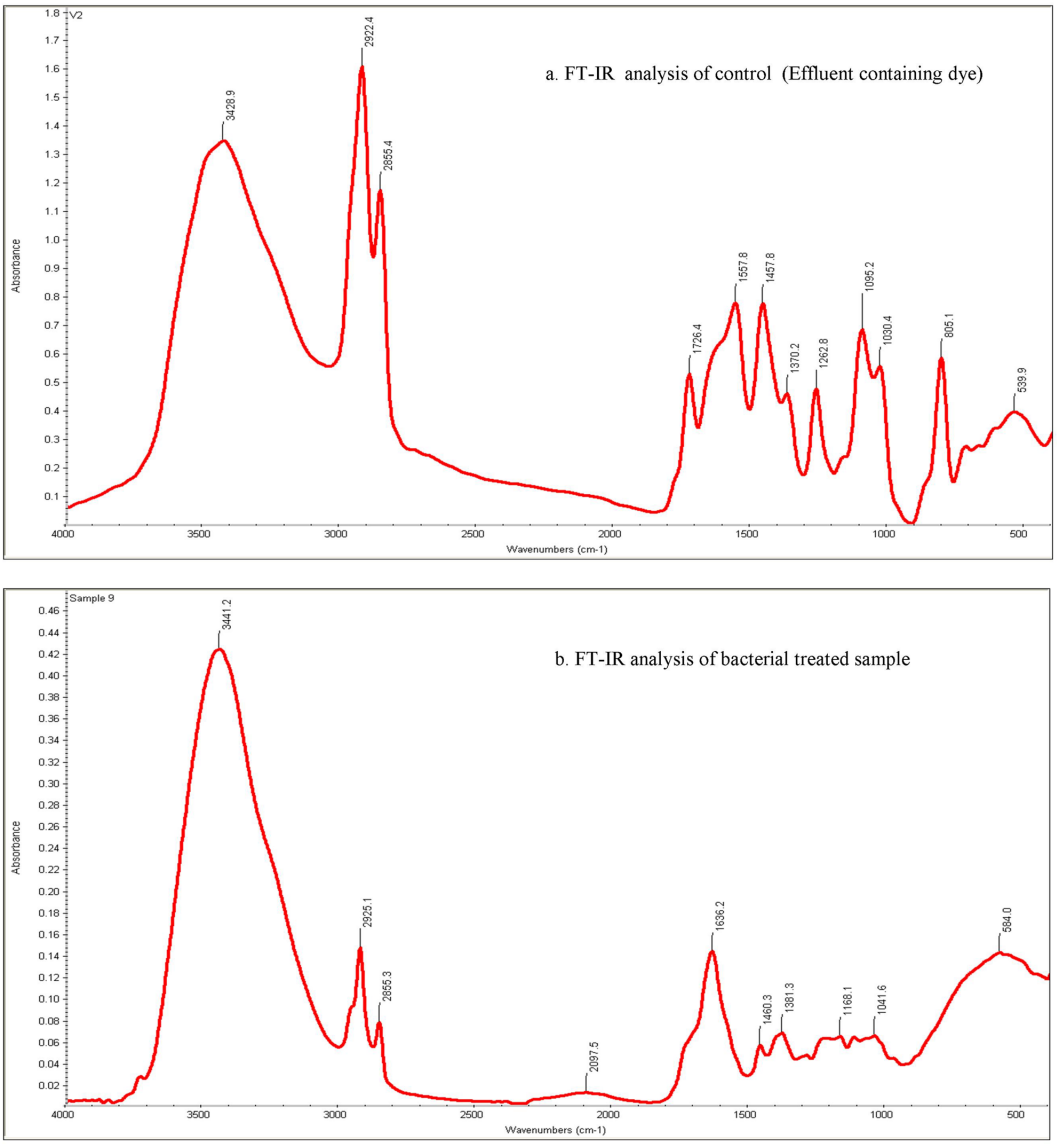
FTIR Analysis of (**a**) Control (Effluent containing the dye) and (**b**) Bacterial treated effluent (20 h).

The FTIR spectrum of the control dye showed the presence of specific peaks at 3428.9 and 2922.4 cm^−1^ due to the presence of azo groups –N=N– stretching. The peak at 1567.8 cm^−1^ corresponds to N–H stretching of N-phenylaniline while the occurrence of peak at 1457.8 cm^−1^ showed S=O stretching of thesulfonic compounds. The disappearance of peaks at 3428.9 and 2922.4 cm^−1^ indicates the reductive cleavage of dye Procion Red H-3B at azo bond position. The peak at1030.4 cm^−1^ indicates the C–H stretching of alkanes while the peak at1636.4 cm^−1^ shows C–H stretching of amines. The untreated sample also showed a severe peak at 805.1 and 539.9 represent the sulphate and nitroso group. The treated sample showed a tapered peak at 3441.2 cm^−1^ demonstrating the stretching vibration of O–H group. Astrong band in the 3000–1500 cm^−1^ range (2925.1, 2855.3, 2097.5 and 1636.2) may match to carbonyl groups in primary amide functions, or it could come into view because of the continuation of C–O stretching of COO−ketonic. The absorption band at 1041.6 and 584.0 cm^−1^ might appear due to aliphatic C–H bending and COO-asymmetric stretching in spectral. The presence of pollutants in the effluent sample were completely absent in the treated effluent sample giving clear confirmation of the dye mineralization. Figure 3 shows the results of FTIR Analysis.

### Study of the organic pollutants by GC–MS analysis

**Figure 4:**
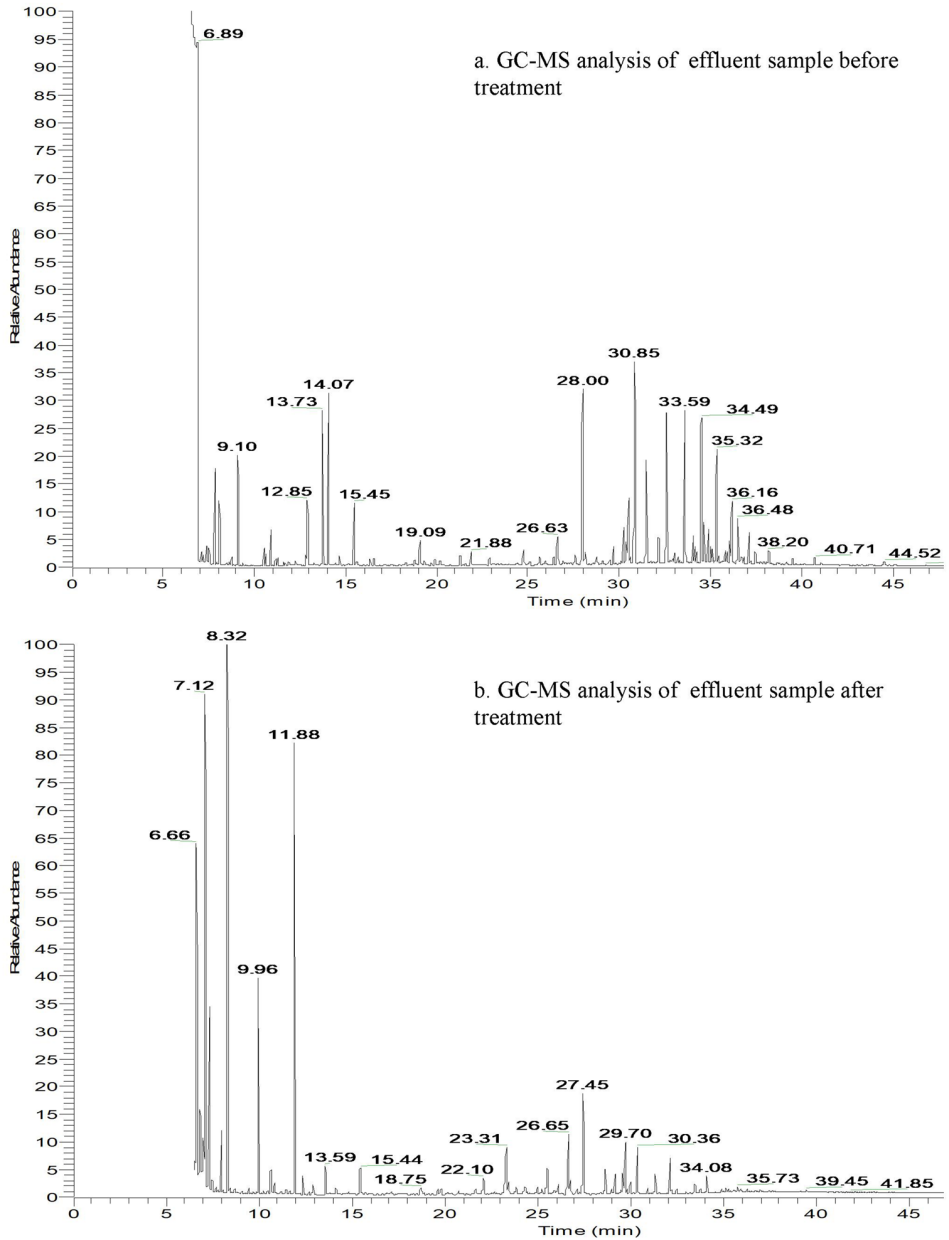
GC–MS analysis of effluent (**a**) before treatment and (**b**) after bacterial treatment.

**Figure 5:**
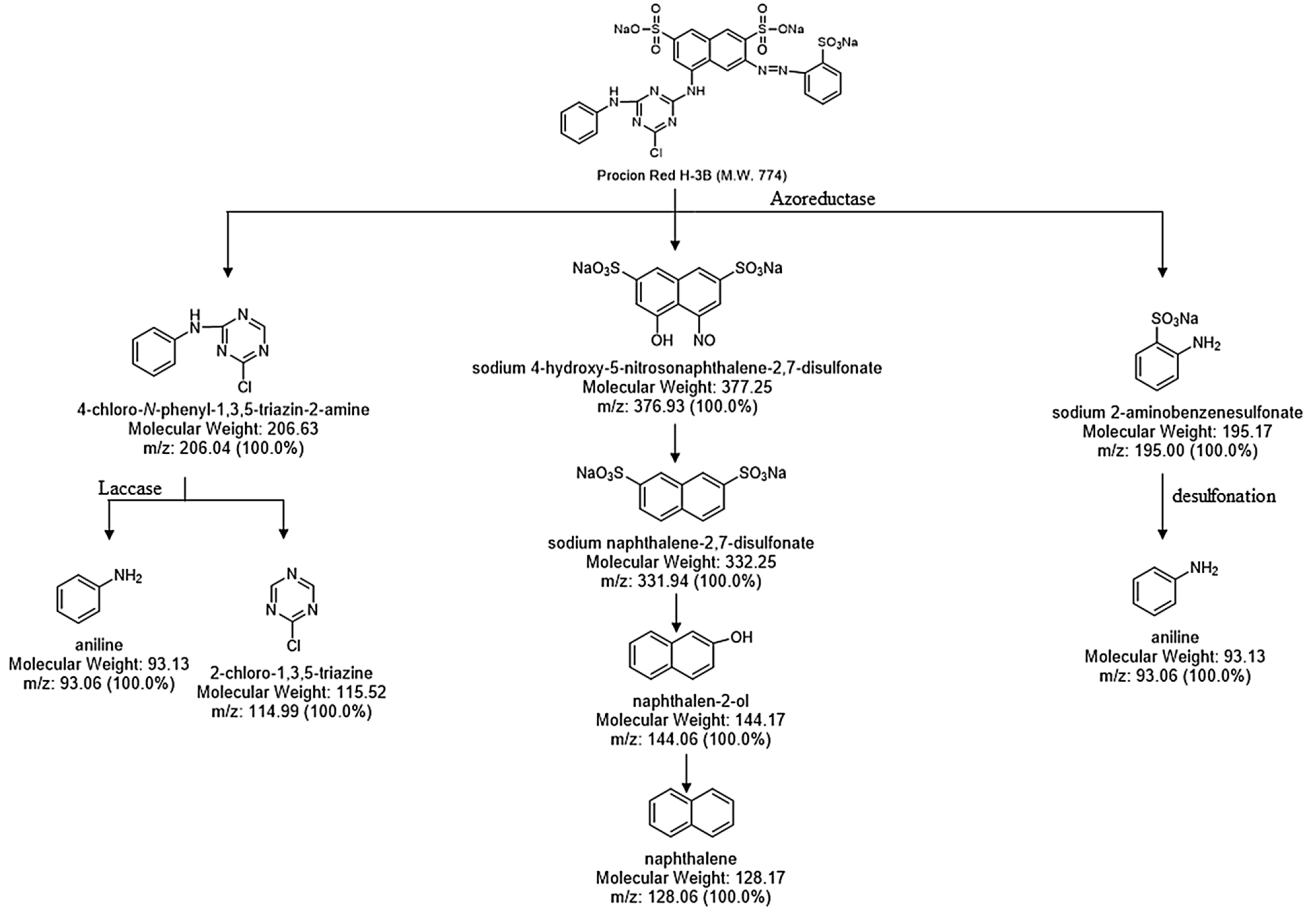
Proposed Metabolic Pathway for degradation of dye Procion Red H3B by *Pseudomonas stutzeri* SPM1.

The enzymatic activity and the GC–MS results helped in proposing a possible pathway for the complete degradation of the dye Procion Red H-3B by the strain *P. stutzeri SPM1*. The GC–MS analysis and enzyme activities suggested the initial reductive cleavage of azo bond leads to the formation of three compounds; (1) 4-chloro-N-phenyl-1,3,5 triazine (MW 169, m/z—206.04), (2) Sodium 4-hydroxy-5-nitrosonapthalene-2,7-disulfonate (MW—377.25, m/z—376.93) and (3) Sodium 2-aminobenzenesulfonate (MW—195.17, m/z—195.00). Which was further broken down to form another three dye intermediates; (1) aniline (MW—93.13, m/z—93.06), (2) 2-chloro-1,3,5triazine (MW—115.52, m/z—114.99), and (3) naphthalene (mw—128.17, m/z—128.06) by the intervention of laccase enzyme before undergoing complete mineralization. Figure 4 shows the result of GC–MS analysis of effluent before and after the bacterial treatment. In accordance with the identified compounds in the GC–MS analysis, a degradation pathway has been proposed in Fig. 5. The organic and inorganic compounds detected in the GC–MS analysis are provided in the Table 4.

### Phytotoxicity analysis

The phytotoxicity of the dye before and after degradation was performed on two agriculturally important plants, *Vigna radiate* and *Cicer arietinum.* 10 seeds from each were sown in separate pots (control, treated and untreated effluent samples). The rate of germination in seeds grown with *P. stutzeri* treated effluent in *Vigna radiate* and *Cicer arietinum* was 80% and 90% respectively. There was no growth in case of *Vigna radiate* seeds when grown with Procion red H-3B dye containing effluent while, minor 10% growth was observed in *Cicer arietinum.* The growth of plumule and radical was significant in both the plants with treated effluent. The data indicates that the bacterial culture has successfully carried the detoxification process. The result is summarised in the Table 3.

**Table 3:** Phytotoxicity study of Procion red H-3B and its degradation product.

Parameters	*Vigna radiata*			*Cicer arietinum*		
	Control( water)	Procion red H-3B	*P. stutzeri* Treated	Control (water)	Procion red H-3B	*P. stutzeri* Treated
Germination (%)	100	0	80	100	10	90
Plumule (cm)	9.45 ± 0.33	0.0***	7.85 ± 0.58	8.56 ± 0.57	2.19 ± 0.25***	7.55 ± 0.50*
Radical (cm)	7.02 ± 1.69	0.0 ***	4.53 ± 0.41**	3.97 ± 0.63	1.05 ± 0.23***	4.25 ± 1.05

Values are mean of three experiments, SD ( ±), significantly different from the control (seeds germinated in water) at *P < 0.05, **P < 0.01, ***P < 0.001 by ANOVA with Tukey–Kramer comparison test a 500 ppm concentration.

## Discussion

In the current study two major changes were made to fasten the decolourization process. First was the replacement of mineral salt medium by phenanthrene enrichment medium for optimizing the *pseudomonas* strain. The ability to degrade multiple PAHs helps the strain achieve enough capability to cause complete mineralization of the dye molecule^12^. Previous research has suggested that the presence of aromatic rings in dyes profoundly affects the impact of oxidation^13^. The second change was carrying on the decolourization study in microaerophilic condition. Decolourization typically occurs in facultative anaerobic condition, because of the lower redox potential of the system^14^. This occurrence may be due to both the electron-withdrawing nature of the azo bond and their resistance to oxygenases attack. Since oxygen is a more effective electron acceptor, it has more predilections for reducing equivalents than the azo dye. The isolated *Pseudomonas* strain when grown under aerobic condition showed minimal growth. Previous studies have reported that when oxygen concentration exceeds the air saturation level, reactive oxygen species (ROS), such as superoxide (O_2_^−^) and hydrogen peroxide (H_2_O_2_), builds up as resultant by-product^15^. Inhibition by oxygen is because of restoration of the oxidized form of flavins. The oxygen toxicity effect thus affects the growth rate significantly.Few research studies correlated the level of colour removal with the dye class rather than with the molecular features^16^ (Table 4).

**Table 4:** Identified organic compounds from untreated effluent (control) and bacterial treated effluent by GC–MS analysis.

S. no	Identified compounds	Retention time	Control	Bacterial treated
1	3-Hydro-4 Nitroso-2,7-Napthalene sulfonic acid	7.12	+	+
2	4-chloro-N-phenyl-1,3,5-triazin-2-amine	7.23	+	+
3	sodium 4-hydroxy-5-nitrosonapthalene-2,7-disufonate	7.86	−	−
4	sodium 2-aminobenzenesulfonate	8.11	−	−
5	3-amino benzene sulfonic acid	8.32	+	+
6	Octadecanoic acid, ethyl ester	8.76	−	−
7	Quinolin-4(3H)-one	9.11	+	−
8	Ethyl tetacosanoate	9.54	−	−
9	N-phenyl aniline	9.96	+	+
10	2-Monostearin, TMS ether	10.13	−	−
11	Napthelin disulfonic acid	11.88	+	−
12	1,1-Biphenyl-4-amine	12.59	−	−
13	11-trans-octadecenoic acid	12.85	−	−
14	1,2-dihyrdonatphtalene	13.59	+	+
15	1,2- Benzenedicarboxylic acid	13.73	−	+
16	Undecenoic acid	14.07	−	−
17	1-Monolinoleoylglycerol trimethylsilyl ether	14.87	+	−
18	2-hydroxy benzene aldehyde	15.44	+	−
19	2,4-Bis(1,1-dimthylethyl)- phenol	15.45	−	−
20	napthalene-2-ol	16.93	+	+
21	1,2,3,4-tetrahydronapthalene-2,3 diol	17.21	−	−
22	N-phenyl-4-quinoneimine	18.75	+	−
23	1-Monolinoleoylglycerol trimethylsilyl ether	19.09	+	−
24	Octadecanoic acid	19.22	−	−
25	2-hydroxy-2H- chromene-2- carboxylic acid	20.34	+	+
26	5-Methyl-1,3-diazaadamantan-6-one	21.88	−	−
27	4-(2-hydroxyphenyl)-2-oxobutanoic acid	22.01	−	−
28	sodium napthalene-2,-disulfonate	23.14	+	−
29	Ethyl[(4-oxo-3,4-dihydroquinolin-2-yl)methyl]acetaldehyde	26.63	−	−
30	napthalene	26.65	+	+
31	Hexadecane, 2,6,11,15-tetramethyl	27.45	−	−
32	Methyl 19-methyl-eicosanoate	27.98	+	−
33	1-sulphonic,2-(4-aminobenzenesul-phonyl) ethanol	28.01	−	−
34	Heptacosane	29.12	+	−
35	Butane-1,3-diol, 1-methylene-3-methyl, bis(TMS)ether	29.88	−	−
36	2-napthol	29.94	+	+
37	Tetratetracontane	30.11	−	−
38	Nonanoic acid	30.21	−	−
39	1,2 dehydroxy naphthalene	30.36	+	+
40	Tetradecanoic acid	30.45	−	−
41	9,12-Octadecanoic acid(Z,Z)-, TMS ester	30.85	+	−
42	8-amino naphthol 3-sulfonic acid	32.15	−	−
43	9-Hexadecanoic acid	32.21	+	−
44	1,2-Benzenedicarboxylic acid, diisooctyl ester	32.81	+	−
45	2-Hexadecanol	33.59	−	−
46	N-(4-bromo-n-butyl)-2-piperidinone	33.84	−	−
47	2,hydroxybenzaldehyde	34.08	+	+
48	2-chloro-1,3,5 triazine	33.98	+	+
49	3,11-Tetradecadien-1-ol	34.59	−	−
50	2-hydroxybenzoic acid	34.08	−	−
51	Propane, 1,2,3-tris[(tert-butyldimethylsilyl)oxy]	34.62	+	−
52	salicylate	35.31	+	+
53	pyruvate	35.32	+	+
54	1-sulphonic,2-(4-aminobenzenesul-phonyl) ethanol	35.73	−	−
55	1,2-Benzenedicarboxylic acid, mono(2-ethylhexyl)ester	36.15	+	−
56	2-Acetyl-3-(2-cinnamido)ethyl-7-methoxyindole	36.16	−	−
57	Methylenebis (2,4,6-triisopropylphenylphosphine)	36.48	−	−
58	Aniline	37.11	+	+
59	3-oxopentanidioc acid	38.2	+	+
60	pyrocatechol	38.4	+	+
61	4-(2-hydroxyphenyl)-2-oxobutanoic acid	39.01	−	−
62	Cyanuric acid	39.33	+	+
63	Acetyl CoA	39.45	+	+
64	β-ketoadipic acid	40.32	+	+
65	Ethyl tetacosanoate	40.71	+	−
66	Succinate	41.45	+	˖

The enzymatic mechanism for degradation of azo compounds by bacterial cell is improved by the occurrence of electron-donating methyl and methoxy substituents and worsened by the presence of electron withdrawing chloro, fluoro, and nitro substituents^17^. The strain possessed azoreductase enzyme which is known to activate direct oxidation of azo dyes by highly reactive electrophilic diazonium salts. Azoreductases are flavin-containing group of enzymes that uses nicotinamide adenine dinucleotide or nicotinamide adenine dinucleotide phosphate as a reducing equivalent^18^. Previous genomic studies of the *Pseudomonas spp.* have highlighted the presence of genes responsible for the production of laccase and NADH-DCIP reductaseenzymes which equally contributes to the effective decolourization and degradation process by the strain. These enzymes are monomeric flavin-free azoreductases, as well as capable of using NADPH and NADH as cofactors in reductive cleavage of numerous sulfonated azo dyes^19^. Our findings were similar to the recent studies reported by Sarkar et al.^20^. The oxidative degradation of aromatic compounds involves the participation of mono and dioxygenases^21^. The identification of pyrocatechol in the GC–MS analysis confirmed the cleavage of azo bond through the ortho pathway. The strain showed significant capability in degrading naphthalene, aniline and 2-chloro-1,3,5triazine. The possible explanation of this can be drawn from previous studies of *Pseudomonas stutzeri* strains where the presence of two characteristic genes cat A and nah H was reported^22^. The gene cat A codes for the enzyme catechol 1,2-dioxygenases; which is responsible for ortho cleavage of catechol. While the gene nah H codes for the enzyme catechol 2,3-deoxygenase which helps in the meta cleavage of catechol which encodes degradation of naphthalene^23^. The presence of many peaks in the GC–MS indicated the presence of degraded dye metabolites. Further the mass spectrum showed the presence of very few peak at retention time 45 min. The major organic compounds detected in untreated sample were organic acids, benzene rings and napthalene compounds. It also had a major proportion of cholro, sulphate and sodium compounds. The compounds that were found in the treated effluent sample were much less toxic than the compounds that were found in the untreated sample. This indicates that the complex and high molecular weight carrying dye compound was degraded by the *Pseudomas stutzeri* strain SPM-1 with the help of bacterial enzyme azoreductase, laccase and NADH-DCIP reductase^5^. The strain was capable to utilise the dye compounds, its intermediates and the resultant metabolites as sole carbon and nitrogen source, thus causing significant role in the decolourization and degradation.

## Materials and method

### Chemicals and reagents

The textile azo dye Procion Red—H3B (CI No. 31701; molecular formula—C_25_H_15_ClN_7_Na_3_O_10_S_3_; molecular weight—774 g/mol) was gently gifted by Rama International textile Ltd. (Surat, Gujarat, India). For the enzyme assay, nicotinamide adenine dinucleotide (NADH), dichloro phenol indophenol (DCIP), phenanthrene enrichment medium, guaiacol (Sigma-Aldrich, USA), ABTS (2, 20-azino-bis (3-ethyl benzothiazoline- 6-sulfonic acid)) and all other chemicals were purchased from Merck KGaA, Darmstadt, Germany.

### Collection and analysis of effluent sample

The effluent sample was collected from the dumping sites of a textile dye manufacturing industries near Pandesara area of Surat, Gujarat, India. The city is one of the leading manufacturers of textile and dyestuffs. Sampling was done by the standard spot and grab procedure from the centre point of the area. Temperature and pH were noted immediately using laboratory thermometer (Mextech DT-9 Digital Thermometer) and pH meter (Digital Hand-Held HI98128 pH meter) respectively. It was then brought in the laboratory and stored at 4 °C for further processing. The physico-chemical parameters (i.e. color, pH, BOD, COD, TDS, etc.) of the textile effluent sample were tested as per standard methods (APHA 2012).

### Optimization of the strain *P. stutzeri* SPM-1

The isolation and screening of the bacterial strain from textile wastewater dumping sites of Surat, Gujarat, India has been previously reported^24^. The isolated *Pseudomonas* species was submitted to the NCBI, GenBank with accession number MW219251. Before proceeding for the decolourization studies the strain was regularly enriched in phenanthrene mineral medium supplied with 2% nitrate and succinate as growth media. The strain was kept for overnight growth by transferring a loopful of bacterial stock culture into a 250 mL Erlenmeyer flask containing 100 mL of enrichment medium at 37 °C for 24 h under shaking condition (120 rpm). All decolourization studies were performed using this overnight grown culture as inoculum.

### Optimization for decolourization and degradation of Procion Red—H3B

To study the effect of different nitrogen sources like yeast extract, urea, meat extract, peptone and malt extract (3 g/L) was added into the bacterial culture medium. The effect of nitrogen source was studied over a period of time (0–120 h) at 50 mg/l dye concentration, 20% inoculums concentration and temperature 37 °C under static conditions. In another experiment, keeping the parameters constant the effect of glucose, maltose, fructose, mannose, starch, lactose, sucrose (10 g/L) was analysed for effect of various carbon sources. Decolourization and degradation of Procion Red—H3B at diverse concentrations viz. 0.1–1.0 (w/v) was carried to optimize the effect of carbon and nitrogen sources.To evaluate the effect of different environmental conditions the same experiment was carried out at different temperature (25–50 °C), pH (4–12), and dye concentrations (50–250 mg/L).

### Decolorization experiment

The decolourization experiments were performed using 250 ml Erlenmeyer flask containing 100 ml of pre-cultured strain *P. stutzeri* SPM-1. The textile dye effluent consisting of the textile azo dye Procion Red—H3B (50 mg/l) was diluted up to 70% and 50 ml of this effluent was added to the flask. The culture containing phenanthrene mineral medium was supplemented with fructose and peptone at a concentration of 0.5% (w/v) and 1.0% (w/v) respectively. The flasks were then incubated in shaker at 150 rpm. The aromatic hydrocarbons formed throughout the course of degradation were estimated by taking 3 ml of culture supernatant at regular interval of time (3 h). Each time the samples were centrifuged at 6000 rpm for 15 min, 4 ± 0.2 °C and the suspended particles were removed by adding equal volume of methanol. The decolourization was examined by measuring the maximum absorbance wavelength between the range 200 and 700 nm at room temperature. The flask without the dye or bacterial culture was kept as control flask and tested under the same conditions. All the experiments were carried in triplets and the average value was noted for calculations. The decolourization percentage was calculated by the following formula:

$${\text{Decolorization }}\left( \% \right) = \frac{{Initial\,absorbnce\left( {Ao} \right) - Final\,absorbance \left( {Ai} \right)}}{{Initial\,absorbance \left( {Ao} \right)}} \times 100$$

### Preparation of cell free extracts

To study the enzyme activity, 20 ml of the pre-enriched strain was inoculated into 80 ml phenanthrene enrichment medium and kept for 24 h incubation. To extract the cell pellets, the culture was centrifuged at 7000×*g* for 15mins and was measured as a control for determining the cells enzymatic activities. The cell biomass were then suspended in 50 mM potassium phosphate buffer (pH 7.4) and sonicated with 7 strokes, each one of 60% amplitude for 30 s at 2 min interval and 4 °C temperature. The same procedure was done for quantification of the enzyme activity after removal of colour from the effluent sample.

### Estimation of enzyme activity

The enzymatic activity during the degradation of dye is studied by processing the bacterial degraded supernatant that was extracted earlier. The laccase activity was estimated by using guaiacol (Sigma-Aldrich, St. Louis, MO, USA) as explained previously^25^. The azoreductase activity was examined as described earlier^26^. NADH-DCIP reductase action was confirmed by method reported earlier using extinction coefficient of 19 mM cm^1^^27^. Enzyme activities were quantified using extinction coefficient of oxidized ABTS (3.6 × 10^4^ M^−1^ cm^−1^) at 420 nm and of methyl red (23,360 M^−1^ cm^−1^) at 430 nm. All enzyme activity experiments were performed in triplicate and average rates were calculated. The expression of enzyme activity is done as international unit (IU).

### Detection of dye mineralization

Reduction in the biological oxygen demand (BOD), chemical oxygen demand (COD) and total organic carbon (TOC) is a clear indication of mineralization of the dye Procion Red -H3B. The cell biomass was extracted by centrifuging both control and decolourised culture broth at 7500×*g* for 15 min at 4 ± 0.2 °C, followed by filtration using 0.45 μm cellulose acetate filters (Sterlitech Corporation, USA). BOD was examined using photometric method. Dichromate closed reflux titrimetric method was used to analyse the reduction in COD, and TOC was estimated using a Sievers 5310C automated analyzer (GE Water & Process Technologies, USA).

### UV–Vis and FT-IR spectroscopic analysis

The bacterial treated as well as untreated culture medium was analysed through a UV–Vis spectrophotometer in the wavelength range between 200 and 600 nm at room temperature^28^. Additionally, FT-IR analysis of the sample was carried in range of 400–4000 cm^−1^ using a spectrophotometer (Nicolet 6700, Thermo Scientific, USA) in order to reveal the chemical nature of the treated effluents. The separated samples of bacterial treated effluent were mixed with potassium bromide (KBr) to prepare the pellet for FT-IR analysis^29^.

### Extraction and identification of organic compounds

The organic compounds that include the dye intermediates and metabolites formed during the degradation of the textile effluent were extracted with equal volume of ethyl acetate under acidic condition as described earlier^30^. Extracts were then dehydrated by evaporation and residual was re-dissolved in HPLC grade methanol for HPLC and GC–MS analysis.

### HPLC analysis

HPLC study was performed using waters, 515 HPLC instrument attached with a diode array detector system (1100 series, Agilent Technologies, USA) and reverse phase C18 column (250 × 4.6 mm, 5 µm particle size) by using the gradient of solvent A (Milli-Q water) and solvent B (acetonitrile with 0.1% TFA) (Merck, Germany) at a flow rate 0.4 mL min^−1^ for 60. To access the decolourization and degradation of Procion Red H-3B by the strain *P. stutzeri* SPM-1, the detection of wavelength was monitored at wavelength 250 nm (absorption maxima). A glass microsyringe (Agilent Technologies, USA) was used to inject methanol extract (50 µL) into the column.

### GC–MS analysis

The derivatization of the extracted samples was carried out using trimethylsilyl (TMS). GC–MS (Trace GC Ultra Gas Chromatograph, Thermo Scientific, USA) equipped with a TriPlus auto sampler coupled to TSQ Quantum XLS triple quadrupole mass spectrometer (Thermo Scientific, USA) was injected with 2.0 µL aliquot of the derivatised sample. DB-5 MS capillary column (30-m length × 0.25 µm I.D. × 0.25 mm film thickness of 5% phenyl and 95% methylpolysiloxane) was used for the separation process. Helium was the carrier gas with a flow rate of 1.1 mL min^−1^. The temperature for the GC oven varied starting at 65 °C (hold for 2 min) to gradually increasing up to 230 °C at the rate of 6 °C min^−1^. The final temperature recorded, reached to 290 °C (hold for 20 min) at a rate of 10 °C min^−1^ high.

For the mass spectrum the positive electron ionization (+ EI) mode at electron energy of 70 eV with a solvent hold-up of 7 min was followed for the operation. A full-scan mode from m/z, 45–800 was used for the operation of mass spectrum. MS library NIST v. 1.0.0.12 was used to detect and identify the organic compounds.

### Phytotoxicity experiments

Phytotoxicity test was carried using *Vigna radiata* (Mung bean) and *Cicer arietinum* (Bengal gram). 10 seeds from each species were grown separately in three set. 10 ml of dye effluent (500 ppm), 10 ml of degradation products (500 ppm) and control set with tap water was added every day to the plants. After 7 days, germination (%) and length of plumule and radical was examined.

### Statistical analysis

The data were examined by one-way analysis of variance (ANOVA) using the Tukey–Kramer multiple comparisons test.

## Conclusion

The ever growing population requires proper management of wastewater. Urbanization has limited the expansion of water bodies leading to enormous scarcity of cleaner usable water. Textile industry, being a part of the process is largely affecting the water quality all over the world. These circumstances are forcing the scientists and environmentalist to look for an alternative source for recycling and reusing the industrial wastewater for various other purposes. Microorganisms which are the natural degraders within the environment will be promising future for the bioremediation of industrial waste waters. This study gives an insight into the successful bioremediation of textile effluent containing Procion Red H-3B. In summary, we effectivelymineralised the textile azo dye Procion Red H-3B using *Pseudomonas stutzeri* SPM-1. The optimization conditions showed immense growth in the decolourization and degradation capability of the *Pseudomonas* strain. These conditions revels its importance for the treatment of textile wastewater. All the quantitative test results proved that the mineralised compounds were safe and non-toxic to the environment. The isolated strain shows great potential in future application forthe treatment of textile wastewater.
